# Fibre Refractometry for Minimally Invasive Sugar Content Measurements within Produce

**DOI:** 10.3390/s24196336

**Published:** 2024-09-30

**Authors:** Mark A. Zentile, Peter Offermans, David Young, Xu U. Zhang

**Affiliations:** IMEC the Netherlands, OnePlanet Research Center, Bronland 10A, 6708 WH Wageningen, The Netherlands

**Keywords:** fibre-optic sensors, refractometry, chemical sensing, postharvest quality, smart agriculture

## Abstract

A minimally invasive needle refractometer is presented for sugar content measurements within produce. A passive sampling cap structure was developed that improves the reliability of the device by avoiding interfering back reflections from the flesh of the produce. It is explained that factory calibration may not be needed for this type of refractometer, potentially reducing production costs. Also demonstrated is an iterative method to correct for temperature variations without the need for an integrated model for how the refractive index changes with temperature for different levels of sugar concentration. The sensor showed a typical standard deviation of 0.4 °Bx for a 10-s-long measurement and was validated against a prism refractometer, showing an average offset of (0.0±0.1) °Bx. In addition, the potential for using the device to investigate sugar distributions within a single fruit sample is demonstrated.

## 1. Introduction

The refractive index of juices is important to measure for many purposes in the agricultural and food industry. The refractive index, along with the juice temperature, is used to calculate a quantity known as the ‘soluble solid content (SSC)’, sometimes also referred to as the total soluble solids (TSS). The SSC of a juice is defined as the weight percentage of dissolved solids for a pure sucrose solution with an equivalent refractive index and temperature to the juice. For most produce, the SSC is driven mainly by sugars and therefore correlates with sweetness and can be used to determine ripeness. Also, partly due to the ease of measurement, SSC is one of the standard parameters for determining produce quality [1].

The standard technique to measure SSC in produce is to mechanically extract the juice from a fruit and to place a drop on the measurement window of a prism refractometer [1]. However, the sample preparation can be somewhat time-consuming, and it is a destructive method. Fast and non-destructive methods have been demonstrated based on millimetre-wave reflectometry [2,3], VNIR spectroscopy [4], VNIR hyperspectral imaging [5,6], FT-IR spectroscopy [7], fluorescence spectroscopy [8] and fluorescence hyperspectral imaging [9], to name a few. Since these methods are sensitive to a large number of concomitant factors (size, shape, surface roughness, etc.), they require extensive calibration with a large data set of representative produce and complex multivariate chemometric techniques. This process is not only complex and time-consuming but also may result in models that are only valid for one cultivar [10] and degrade from one season to the next [11]. Techniques have been developed to update these calibration models with a minimal amount of further effort [12]; however, they cannot recover the near calibration-free convenience and robustness of refractometry.

This study presents a technique that is non-destructive whilst maintaining the advantages of refractometry, namely, a minimally invasive refractometer realised through a thin optical fibre in a needle format. Also, this format allows highly localised measurements. In this manner, sugar distributions within a single fruit can be measured. We pay particular attention to demonstrating that the technique can be made into a practical, low-cost device through simple Fresnel reflection-based refractometry [13]. The common alternative to Fresnel reflection refractometry is the use of a resonant structure along with a wavelength interrogator [14,15,16]. While this can give excellent measurement precision, the use of a wavelength interrogator means that this technique can be costly. On the other hand, Fresnel reflection refractometers often require a reference fibre [17,18,19,20] to correct for either light source intensity fluctuations or detector sensitivity drifts. This increases the complexity and cost of a device. We show sufficient accuracy and precision without the need for a reference fibre. Also, the consistency of fibre refractive indices means this type of refractometer does not require an initial calibration, which is usually performed by fitting a calibration curve to sucrose solutions [21]. This again lowers the cost of the envisaged device by removing the need for a factory calibration step. The only calibration required is a single measurement of air (generally the most easily available reference sample), after which the device can measure the SSC in produce at varying (known) temperatures. Furthermore, the choice to work at a wavelength of 1550 nm allows the device to use low-cost, high-quality lasers and fibre-optic components from the telecommunications industry. Overall, we expect that a product based on the device will cost only twice that of a digital prism refractometer whilst being approximately 5 times less expensive than fibre sensors based on resonant structures probed with low-cost on-chip wavelength interrogators [22].

However, one practical issue of using this type of refractometer for a sample containing solid structures (i.e., flesh) is that back reflections cause significant errors in the measurement, which must be mitigated. Therefore, we have developed a cap structure for the fibre probe that eliminates back reflections. Other techniques to deal with back reflections have been developed for incoherent light [23]; however, a minimally invasive needle probe motivates the use of a coherent light source. This is because thin optical fibres must be used in thin probes, and therefore, a low-etendue light source, namely, a laser, is required to launch sufficient light into the fibre. Laser light will typically have a coherence length much longer than the structures in fruit flesh.

## 2. Theory

### 2.1. Measuring Refractive Index from Fresnel Reflection

The Fresnel reflection coefficient of normal incident light at an interface of an optical fibre is given by the well-known formula

(1) $$R\equiv \frac{I}{I_{0}}={\left(\frac{n_{s}-n_{f}}{n_{s}+n_{f}}\right)}^{2},$$

where *I* is the reflected intensity, $I_{0}$ is the incident intensity, $n_{f}$ is the effective refractive (phase) index of the optical fibre, and $n_{s}$ is the refractive index of the sample beyond the facet. For fibre refractometry, the sample refractive index, $n_{s}$, is determined through the measurement of *I*, given a known value of $n_{f}$. Therefore, we re-write Equation (1) as

(2) $$n_{s}=n_{f}\left(\frac{1-\sqrt{I/I_{0}}}{1+\sqrt{I/I_{0}}}\right).$$

Note that since Equation (1) is symmetric around $n_{s}=n_{f}$, there is ambiguity as to whether the sample refractive index is higher or lower than $n_{f}$. However, for fruit juice sugar concentration measurements, $n_{s}$ is usually lower than $n_{f}$; therefore, we select this form in Equation (2).

One additional complication is that $I_{0}$ is not directly known and must be determined through the measurement of a sample with a known refractive index, e.g., air. If the fibre refractive index is also unknown, both $n_{f}$ and $I_{0}$ can be determined through an additional measurement of another sample with a known refractive index [24]. In our case, we used a Corning^®^ SMF-28^®^ fibre with light at a wavelength of 1550 nm, where the phase index is already known, $n_{f}=1.44961$ [25]. Note that due to the small numerical aperture of the fibre, it is a good approximation to assume a normal incident angle for all incident light at the interface of the optical fibre.

### 2.2. Relating Refractive Index and Temperature to SSC

To the best of our knowledge, there is no reported formula to relate the refractive index of a sucrose solution at 1550 nm to SSC at varying temperatures. However, ref. [26] does provide data that can be used to give the sucrose mass fraction as a function of the refractive index of 1550 nm light, but only at a temperature of 298 K:

(3) $$w\left(n\right)=C_{3}{\left(n-n_{w}\right)}^{3}+C_{2}{\left(n-n_{w}\right)}^{2}+C_{1}\left(n-n_{w}\right),$$

where the constants *C*_1–3_ and $n_{w}$ are given in Table A1 in Appendix A.

Temperature changes cause changes in density through thermal expansion. The change in the number density of molecules causes the refractive index to change. This effect does not alter the SSC, and thus, Equation (3) gives an increasing error as the sample temperature deviates from a temperature of 298 K. To correct for temperature differences, the measured refractive index value needs to be scaled to the value that would have been measured at 298 K. This can be realised through the relationship between the (temperature-dependent) density of the sample and its refractive index. The Lorentz–Lorenz equation relates the refractive index of a substance to its number density (*N*) multiplied by its polarisability (α):

(4) $$\frac{n^{2}-1}{n^{2}+2}=\frac{4\pi }{3}N\alpha .$$

In a mixed substance, α is the average polarisability weighted by the mass fraction. If we assume that this polarisability is not altered significantly due to temperature-induced density changes, then we can write

(5) $$\frac{n^{2}-1}{n^{2}+2}\propto \rho ,$$

where ρ is the density of the sample. With an independent way of evaluating the density as a function of *w* and temperature (*T*), we can use an iterative relationship to scale the refractive index. Ref. [27] provides a formula to calculate the density of a sucrose solution as a function of *T* and concentration,

(6) $$\rho \left(S,T\right)=\left(a_{1}+b_{1}S+c_{1}S^{2}\right)+\left(a_{2}+b_{2}S+c_{2}S^{2}\right)T+\left(a_{3}+b_{3}S+c_{3}S^{2}\right)T^{2},$$

where *S* is the concentration in mass/volume percentage, and $a_{i}$, $b_{i}$ and $c_{i}$ are constants relating to the *i*th term and are found by fitting to experimental data. These constants are listed in Table A2 in Appendix A.

When evaluating Equation (6), we make the approximation that S≈w·100 g/cm^3^. This approximation yields an error <3% for concentrations of up to 50 g of sucrose in 100 mL of solution. We then use the following iterative relationship for scaling the refractive index to that which would have been measured at 298 K:

(7) $$\begin{array}{cc} w_{i+1} & =w(n_{i+1}),\\ n_{i+1} & =2n_{0}-\sqrt{\frac{2k_{i}\rho \left(w_{i},T\right)+1}{1-k_{i}\rho \left(w_{i},T\right)}},\\ k_{i} & =\frac{1}{\rho (w_{i},T=298\;K)}\cdot \frac{n^{2}-1}{n^{2}+2}, \end{array}$$

where $n_{0}=n_{s}$, the measured refractive index.

## 3. Materials and Methods

**Figure 1:**
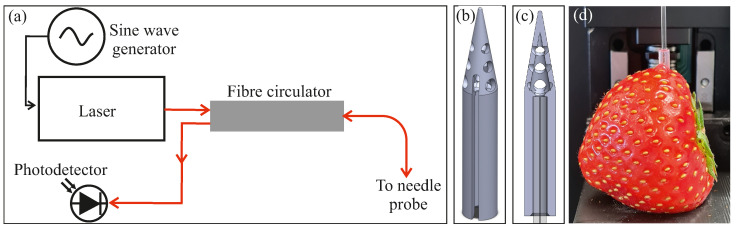
The schematic and design of the experimental apparatus. (**a**) The schematic of the sensing system. A laser launches light into a single-mode optical fibre. The laser intensity is modulated with a sine wave generator. A fibre circulator directs the light to the needle probe, and reflected light is directed by the circulator to a photodetector. (**b**) A rendering of the probe cap design. (**c**) A cutout rendering showing the location of the needle probe when mounted in the cap. (**d**) A photo of the needle probe within the probe cap after insertion into a sample fruit.

Figure 1 shows a schematic of the experimental apparatus used. A fibre-coupled laser (KLS1550, Thorlabs, Dachau, Germany) generated light at 1550 nm, which was power-modulated at 67 Hz (between approximately 0 and 2 mW) by following the signal of a sine wave generator (Analog Discovery 2, Digilent, Pullman, WA, USA). The modulation frequency was chosen to be much less than the bandwidth limit of the laser (3 dB drop at 600 Hz) whilst avoiding powerline interference, which occurs at 50 or 60 Hz, depending on the local mains power supply. The light was coupled into a single-mode fibre and connected to a circulator (6015-3-APC, Thorlabs, Dachau, Germany), which directed the light to the needle probe (Precision Micro-Optics Inc., Burlington, VT, USA). The light reflected from the needle probe re-entered the circulator and was directed to a photodetector consisting of a germanium photodiode with a transimpedance amplifier (PDA30B2, Thorlabs, Dachau, Germany). The photodetector voltage was read out with an oscilloscope (Analog Discovery 2) at 5 kHz over 10 s. The measurement time of 10 s was chosen since this is long enough to keep the measurement precision well below 1 °Bx whilst allowing a similar sample throughput to current handheld refractometers. A measurement of the signal intensity was the result of taking the magnitude of a discrete Fourier transform of the voltage trace at the chosen modulation frequency.

The needle probe consisted of a flat polished single-mode fibre mounted within a glass capillary, with an outer diameter of 1.0 mm, giving the fibre rigidity and allowing it to be pushed into the produce flesh without breaking. This design of the probe alone is not sufficient to give reliable measurements of the refractive index of juice while inside the fruit. This is due to unwanted back reflections from the flesh of the produce, which couple back into the fibre and interfere with the signal due to Fresnel reflection at the fibre end face. This interference can be constructive or destructive, depending on the relative phase of the resulting back reflection, and can therefore cause a corresponding under- or overestimation of the refractive index. To avoid these back reflections, a probe cap was designed that separated the juice from the flesh within the fruit. Panel (b) of Figure 1 shows the cap design used [28]. The cap was manufactured with an SLA 3D printer (Formlabs, Form 3B+). A successful cap design needs to meet several requirements:

1. It should allow the juice to come into contact with the fibre end whilst keeping the flesh outside the cap;
2. The internal walls of the cap need to be at an angle such that reflections from the surface are not sent back to the fibre end within the angle of acceptance of the fibre;
3. To prevent a pocket of air from being trapped at the fibre face, there must be a channel to allow air to escape as the cap is pushed into the produce.

The depth at which the probe cap is pushed into the fruit was found to be important. With the cap design shown in Figure 1b, a depth of more than 5 mm (as measured from the fibre end face) would risk pushing flesh into the cap, causing measurement errors by back reflections.

## 4. Results and Discussion

**Figure 2:**
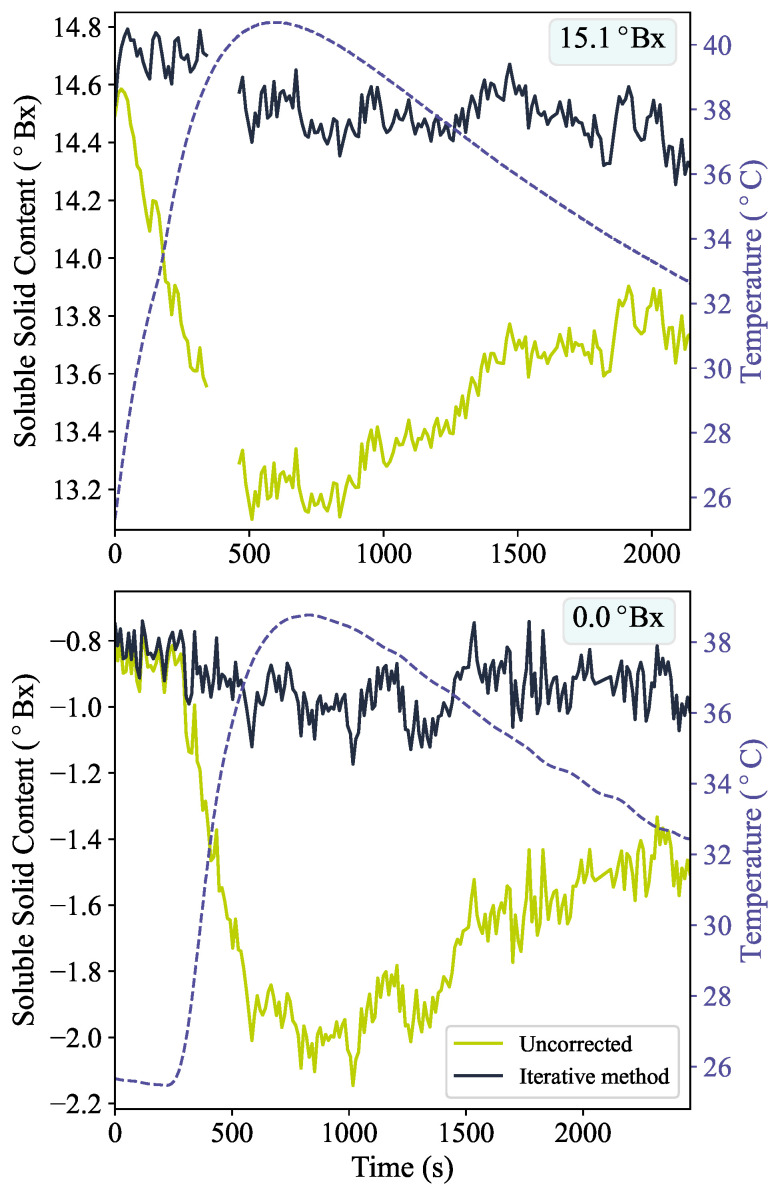
Soluble solid content derived from fibre-based refractive index measurements over time whilst varying the sample temperature. The dashed purple lines show the sample temperature. The solid green lines give the results when using only Equation (4), while the solid dark blue line is the result of applying the iterative temperature correction method. The top panel shows the results for a 15.1 °Bx solution, while the bottom panel shows the results for pure water. The data presented in this figure are available in the Supplementary Material.

The iterative temperature correction method described in Section 2.2 was tested by performing measurements whilst varying the temperature of solutions of known SSC. A hotplate with a magnetic stirrer was used to heat the sample from approximately 25 to 40 °C, after which the sample was allowed to cool. During the temperature cycle, measurements were simultaneously taken with the fibre refractometer and a thermometer. Figure 2 shows the measured SSC and temperature over time for a sample of pure water and a sucrose solution of 15.1 °Bx. The SSC is plotted both without a temperature correction, i.e., using only Equation (3), and with the iterative procedure described in Section 2.2.

It can be seen that without the temperature correction, the SSC deviates by approximately −0.1 °Bx/°C as the sample heats above the standard temperature (25 °C). With the iterative temperature correction method, the SSC remains more constant as the temperature is changed. Also visible is an underestimate of the SSC. The average temperature-corrected SSC is measured to be −0.9 °Bx and 14.5 °Bx, for pure water and the 15.1 °Bx sucrose solution, respectively. The underestimate is attributed to the fibre refractive index being higher than the expected value of 1.44961 due to manufacturing differences. Different probes show small differences in this underestimate, ranging from −1.0 to −0.2 °Bx for measurements of water with 10 different probes. For the probe used to take the data for Figure 2, using a value of 1.4511 for the fibre refractive index gives average temperature-corrected SSC values of −0.2 °Bx and 15.2 °Bx for pure water and the 15.1 °Bx sucrose solution, respectively.

**Figure 3:**
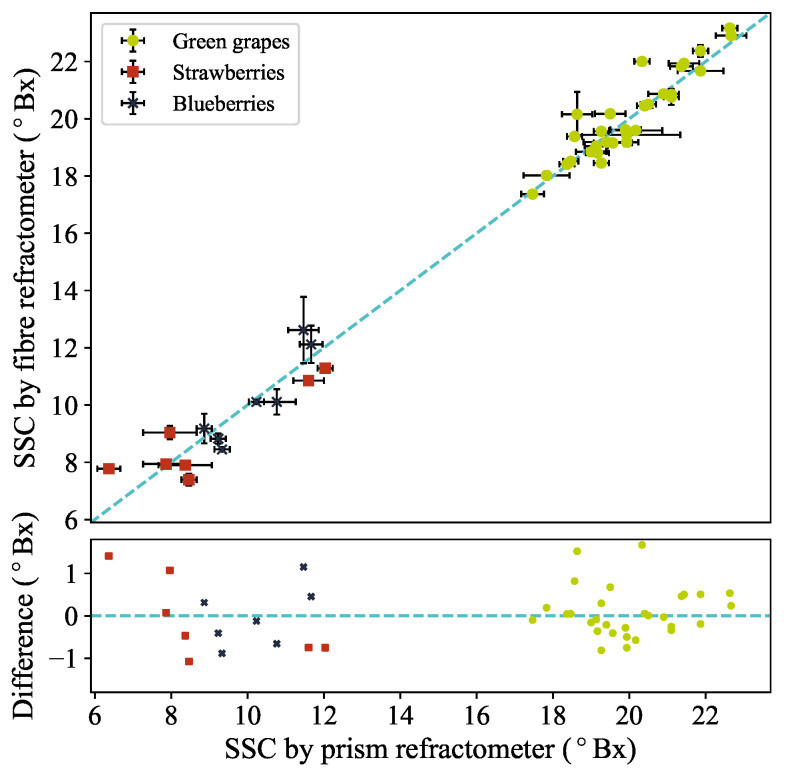
(**Top panel**) Plotted are the SSCs of whole fruit samples measured by the fibre refractometer against measurements by a prism refractometer. (**Bottom panel**) Fibre refractometer response minus the reference prism refractometer plotted against the prism refractometer response. In both panels, the green points, red squares and blue crosses correspond to green grapes, strawberries and blueberries, respectively. The dashed blue line is the line of equivalence. The data presented in this figure are available in the Supplementary Material.

The performance of the device was tested on small fruits. Small batches of strawberries, blueberries and grapes were tested after allowing them to thermalise to the ambient laboratory temperature. The probe end, with a one-time-use 3D-printed cap, was pushed into whole fruits at a depth between 2.5 and 5 mm. For each sample, five measurements were taken in quick succession, after which the juice was squeezed out by hand three times and measured on a digital prism refractometer. Between measurements on each fruit, the cap was replaced and the fibre end was cleaned. Figure 3 shows the average SSC given by the fibre sensor against the average SSC measured with the prism refractometer (HI96801, Hanna Instruments^®^ HI96801, Woonsocket, RI, USA). The measurement uncertainties for the fibre measurements are statistical in nature, whereas the uncertainty in the prism refractometer measurements is likely due to inhomogeneous sugar distribution within the fruits. As such, the error bars shown for the fibre measurements are the standard error, while the error bars for the prism refractometer are given by the full range of the three measurements or 0.2 °Bx (the manufacturer-quoted instrument accuracy), whichever is larger.

The two refractometer measurements show very good agreement. The average difference is (0.0±0.1) °Bx, while the root-mean-square deviation is 0.6 °Bx. This gives confidence that the fibre device can give accurate measurements of the SCC of juice from within a fruit. This is despite the fact that the two measurement techniques have different susceptibilities to differences in the SSC distribution within a fruit, since the fibre sensor measures a much smaller volume of juice.

**Figure 4:**
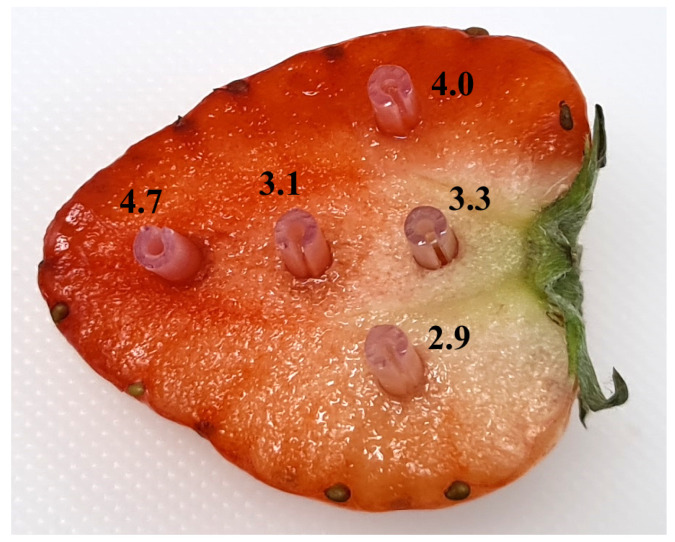
A photograph of a strawberry segment after measurement with the fibre refractometer at five locations. After measurement, the probe cap was left in the fruit to show the location of the measurement. The overlaid numbers are the measured SSC values in °Bx.

The fact that the fibre sensor measures a small volume of juice allows the device to measure the sugar distribution within small fruits in a convenient way, with minimal sample preparation. To demonstrate this, a strawberry was cut in half from peduncle to apex, and the SSC was measured at five locations. Figure 4 shows the result of the measurements.

The measured SSC is highest in the apex and shows back/front asymmetry, as expected from previous studies [29].

## 5. Conclusions and Outlook

We have shown that a simple fibre refractometer based on Fresnel reflection is suitable for SSC measurements within produce. A 3D-printed fibre cap was developed that improves the reliability of the device by avoiding interfering back reflections from the flesh of the produce. We have also demonstrated an iterative method to correct for temperature variations without the need for a per-device calibration measurement. We have seen that the fibre refractometer gives a localised measurement of SSC in produce, which could be used as a convenient method to investigate SSC distributions within a single fruit sample. Whilst the technique may not be practical for high-resolution imaging of SSC distributions, such as that achieved through near-infrared spectroscopy [30], it may still find use as a reference device for model building in these imaging techniques. For the purpose of postharvest quality control, measurements of SSC are based on the average of all the juice within the fruit. Spatial differences may hinder the measurement of this average, so it may help the reliability of the measurements to develop a per-fruit protocol for measurements to handle typical SSC distributions within a fruit. This could be a scheme to average measurements from well-defined sections of the fruit, as is already recommended for prism refractometer measurements [1]. By using thin optical fibres, a minimally invasive device becomes possible. Further work is needed to establish how much the needle puncture affects produce shelf life. It is expected that fruit type, needle cleanliness and puncture size will be important factors to consider. Currently, the full diameter of the probe with the cap is 2.5 mm, but we believe there is scope to reduce the size further, if required.

## 6. Patents

Mark Zentile, Xu Zhang, Peter Offermans, David Young, and Arjan Tibbe. A probe, a system and a method for analysis of a liquid in a mixture of the liquid and solid substance. European patent EP 4 312 017 A1, filed 28 July 2022, US patent US 2024/0035964 A1, filed 20 July 2023.

## Data Availability

The data presented in the study are included in the article or Supplementary Material. Further inquiries for raw data can be directed to the corresponding author.

## Supplementary Materials

The following supporting information can be downloaded at https://www.mdpi.com/article/10.3390/s24196336/s1: Minimal Dataset corresponding to Figures S1 and S2.

## Abbreviations

The following abbreviations are used in this manuscript:

SSC	Soluble solid content

## Appendix A

**Table A1:** Constants corresponding to Equation (3). These constants were found by fitting to data provided in ref. [26].

Constant	Value
$n_{w}$	1.3166
$C_{1}$	7.0700
$C_{2}$	−16.849
$C_{3}$	25.832

**Table A2:** Constants corresponding to Equation (6), taken directly from ref. [27].

*i*	$a_{i}$	$b_{i}$	$c_{i}$
1	1.00042	0.004056	$-4.058\times 10^{-6}$
2	$-2.294\times 10^{-6}$	$-1.332\times 10^{-5}$	$2.661\times 10^{-7}$
3	$-4.646\times 10^{-6}$	$1.254\times 10^{-7}$	$-3.043\times 10^{-9}$

## References

[1] OECD (2018). Guidelines on Objective Tests to Determine Quality of Fuit and Vegetables, Dry and Dried Produce.

[2] Oda M., Mase A., Uchino K. (2012). Nondestructive Measurement of Sugar Content in Apples by Millimeter-Wave Reflectometry. J. Infrared Millim. Terahertz Waves.

[3] Yang Z., Pathak P.H., Sha M., Zhu T., Gan J., Hu P., Mohapatra P. On the feasibility of estimating soluble sugar content using millimeter-wave. Proceedings of the IoTDI ’19: International Conference on Internet-of-Things Design and Implementation.

[4] Walsh K.B., Blasco J., Zude-Sasse M., Sun X. (2020). Visible-NIR ‘point’ spectroscopy in postharvest fruit and vegetable assessment: The science behind three decades of commercial use. Postharvest Biol. Technol..

[5] Wu D., Sun D.W. (2013). Advanced applications of hyperspectral imaging technology for food quality and safety analysis and assessment: A review—Part II: Applications. Innov. Food Sci. Emerg. Technol..

[6] Mishra P., Sytsma M., Chauhan A., Polder G., Pekkeriet E. (2022). All-in-one: A spectral imaging laboratory system for standardised automated image acquisition and real-time spectral model deployment. Anal. Chim. Acta.

[7] Yan-de L., Yi-bin Y. (2004). Measurement of sugar content in Fuji apples by FT-NIR spectroscopy. J. Zhejiang Univ. Sci..

[8] Navrátil M., Buschmann C. (2016). Measurements of reflectance and fluorescence spectra for nondestructive characterizing ripeness of grapevine berries. Photosynthetica.

[9] Noh H.K., Lu R. (2007). Hyperspectral laser-induced fluorescence imaging for assessing apple fruit quality. Postharvest Biol. Technol..

[10] Valente M., Leardi R., Self G., Luciano G., Pain J.P. (2009). Multivariate calibration of mango firmness using vis/NIR spectroscopy and acoustic impulse method. J. Food Eng..

[11] Guthrie J.A., Reid D.J., Walsh K.B. (2005). Assessment of internal quality attributes of mandarin fruit. 2. NIR calibration model robustness. Aust. J. Agric. Res..

[12] Mishra P., Woltering E. (2021). Handling batch-to-batch variability in portable spectroscopy of fresh fruit with minimal parameter adjustment. Anal. Chim. Acta.

[13] Meyer M.S., Eesley G.L. (1987). Optical fiber refractometer. Rev. Sci. Instrum..

[14] Urrutia A., Del Villar I., Zubiate P., Zamarreño C.R. (2019). A Comprehensive Review of Optical Fiber Refractometers: Toward a Standard Comparative Criterion. Laser Photonics Rev..

[15] Cano-Velázquez M.S., Hendriks A.L., Picelli L., van Veldhoven R.P.J., Fiore A. (2023). Temperature-Compensated Solution Concentration Measurements Using Photonic Crystal Fiber-Tip Sensors. Sensors.

[16] Cano-Velázquez M.S., Buntinx S., Hendriks A.L., Van Klinken A., Li C., Heijnen B.J., Dolci M., Picelli L., Abdelkhalik M.S., Sevo P. (2024). Beyond spectral resolution in optical sensing: Picometer-level precision with multispectral readout. arXiv.

[17] Chang K.A., Lim H.J., Su C.B. (2002). A fibre optic Fresnel ratio meter for measurements of solute concentration and refractive index change in fluids. Meas. Sci. Technol..

[18] Su H., Huang X.G. (2007). Fresnel-reflection-based fiber sensor for on-line measurement of solute concentration in solutions. Sens. Actuators B Chem..

[19] Pan J., Huang X., He Y., Huang B. (2012). Fresnel-reflection-based fiber sensor for high-temperature measurement. Rev. Sci. Instrum..

[20] Xu W., Huang X.G., Pan J.S. (2013). Simple Fiber-Optic Refractive Index Sensor Based On Fresnel Reflection and Optical Switch. IEEE Sens. J..

[21] Aronne G., Malara P. (2019). Fiber-optic refractometer for in vivo sugar concentration measurements of low-nectar-producing flowers. New Phytol..

[22] Yebo N.A., Bogaerts W., Hens Z., Baets R. (2011). On-Chip Arrayed Waveguide Grating Interrogated Silicon-on-Insulator Microring Resonator-Based Gas Sensor. IEEE Photonics Technol. Lett..

[23] Zhang X.U., Faber D.J., Post A.L., van Leeuwen T.G., Sterenborg H.J.C.M. (2019). Refractive index measurement using single fiber reflectance spectroscopy. J. Biophotonics.

[24] Zhang X.U., Post A.L., Faber D.J., van Leeuwen T.G., Sterenborg H.J.C.M. (2017). Single fiber reflectance spectroscopy calibration. J. Biomed. Opt..

[25] Kim C.B., Su C.B. (2004). Measurement of the refractive index of liquids at 1.3 and 1.5 micron using a fibre optic Fresnel ratio meter. Meas. Sci. Technol..

[26] Saunders J.E., Sanders C., Chen H., Loock H.P. (2016). Refractive indices of common solvents and solutions at 1550 nm. Appl. Opt..

[27] Darros-Barbosa R., Balaban M.O., Teixeira A.A. (2003). Temperature and Concentration Dependence of Density of Model Liquid Foods. Int. J. Food Prop..

[28] Zentile M., Zhang X., Offermans P., Young D., Tibbe A. (2022). A Probe, a System and a Method for Analysis of a Liquid in a Mixture of the Liquid and Solid Substance. European Patent.

[29] Ikegaya A., Toyoizumi T., Ohba S., Nakajima T., Kawata T., Ito S., Arai E. (2019). Effects of distribution of sugars and organic acids on the taste of strawberries. Food Sci. Nutr..

[30] Sugiyama J. (1999). Visualization of Sugar Content in the Flesh of a Melon by Near-Infrared Imaging. J. Agric. Food Chem..

